# Deep architectures for long-term stock price prediction with a heuristic-based strategy for trading simulations

**DOI:** 10.1371/journal.pone.0223593

**Published:** 2019-10-10

**Authors:** Catalin Stoean, Wiesław Paja, Ruxandra Stoean, Adrian Sandita

**Affiliations:** 1 Romanian Institute of Science and Technology, Cluj-Napoca, Romania; 2 Grupo Ingeniería de Sistemas Integrados (TIC-125), E.T.S.I. Telecomunicación, Universidad de Malaga, Malaga, Spain; 3 Faculty of Mathematics and Natural Sciences, University of Rzeszów, Rzeszów, Poland; 4 Faculty of Sciences, University of Craiova, Craiova, Romania; Liverpool John Moores University, UNITED KINGDOM

## Abstract

Stock price prediction is a popular yet challenging task and deep learning provides the means to conduct the mining for the different patterns that trigger its dynamic movement. In this paper, the task is to predict the close price for 25 companies enlisted at the Bucharest Stock Exchange, from a novel data set introduced herein. Towards this scope, two traditional deep learning architectures are designed in comparison: a long short-memory network and a temporal convolutional neural model. Based on their predictions, a trading strategy, whose decision to buy or sell depends on two different thresholds, is proposed. A hill climbing approach selects the optimal values for these parameters. The prediction of the two deep learning representatives used in the subsequent trading strategy leads to distinct facets of gain.

## 1 Introduction

Stock price prediction has been an evergoing challenge for economists but also for machine learning scientists. Different approaches have been applied over the decades to model either long-term or short-term behavior, taking into account daily prices and other technical indicators from stock markets around the world. During the last years, deep learning has also entered the stock market realm, particularly through its specific technique to model long-term data dependencies, the long short-term memory network (LSTM).

The present paper puts forward a new data set holding long-term data from 25 companies enlisted at the Bucharest (Romania) stock market and appoints a LSTM architecture to predict the subsequent close price. A convolutional neural network (CNN) with 1D (temporal) convolutions is also employed to produce an estimation of the next day close price value. The predictions of the two models are then used in a trading scenario. The difference between the current close price and its estimated value for the following day is measured against two thresholds—depending on which of them is higher—in order to pursue a BUY or SELL action. The values for the two thresholds are found via the heuristic approach of hill climbing (HC). The results—showing different measures of gain from the simulated transactions of shares for the 25 companies—indicate that the two deep learning approaches achieve a different way of learning. Their predictive prices make one excel at the total amount of money gained in the transactions over all companies and the other at the largest number of companies with profit after transactions.

The recent research in the area of stock price prediction with deep learning methodologies makes use mainly of LSTM architectures and takes into account several predictors. In this light, besides putting forward a new data set, the aim of the proposed framework is to show that also 1D CNN can equally and faster help predict the next move of the stock price, as already demonstrated in literature [1] for different sequential tasks. Also, this study shows that there is no need to further complicate the current learning problem with more predictive variables or further feature extraction from these, as demonstrated in comparison to the methodology of a different study in section 3.2.

There is also a practical aim to this study, regarding the subsequent trading simulation on the base of the deep learning predictions. The state of the art either employs a deterministic scheme (the literature entries using deep learning) or very complex evolutionary algorithms for trading rule generation (the papers using other machine learning techniques for prediction). In opposition, the approach presented in this paper parametrizes the BUY and SELL rules and determines the optimal variables through a simple HC heuristic.

The paper is structured in the following manner. Section 2 begins with the description of the novel data collection and draws the conceptual premises for the subsequent modelling. The chosen deep architectures and the proposed heuristic-driven search strategy are outlined against the state of the art. The experimental part, found in section 3, is composed of the exploration of the best parameter settings, the results of the two deep models and the effect of their predictions within the HC-powered trading strategy on the hypothetically generated profit. The discussion is concluded in section 4, by also advancing directions for further improvement.

## 2 Materials and methods

The new data employed in this study is described in detail both as concerns its content and the means to access it. The methodology is outlined with respect to the state of the art in deep learning for stock price prediction. The trading approach driven by the estimations of the deep learners and the parametrization of HC is presented in comparison to the classical strategies of buy & hold and Bollinger bands.

### 2.1 Stock price data from the Bucharest Stock Exchange

**Fig 1:**
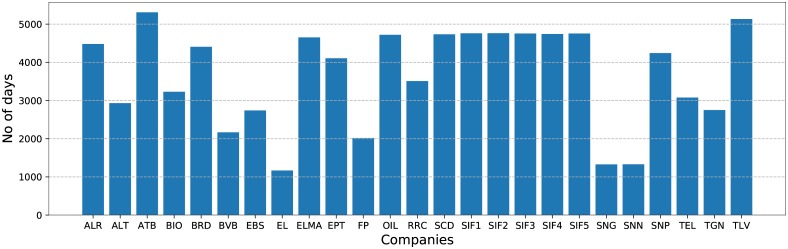
The number of days available for each company in turn.

**Fig 2:**
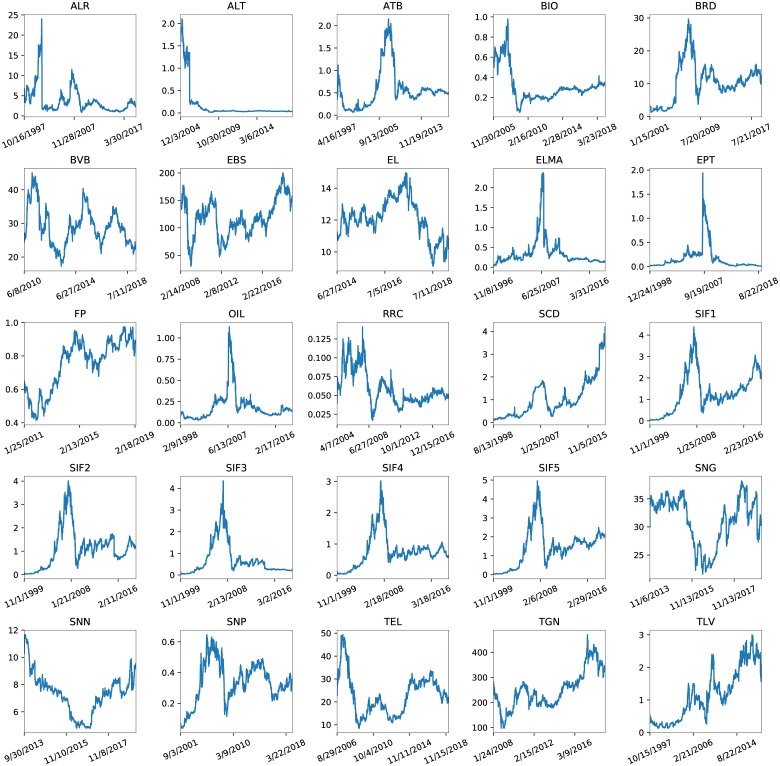
The close prices on the vertical axes along the recorded period on the horizontal ones for each company in turn. The date format used is month/day/year.

The data used refers 25 companies listed under the Romanian stock market. The trading indicators are the number and value of transactions, the number of shares, the minimum, average and maximum prices, the open and close price. The data has been collected since October 16 1997 until March 13 2019. The period for which each business is listed is different, as triggered by the date of the enlisting of the company, as well as the cease of activity at the other end. Fig 1 shows the available history for each of them. The overall trend for the close price as well as an indication of the periods in which this was recorded are illustrated in Fig 2. The collection is publicly available at the following link: https://doi.org/10.6084/m9.figshare.7976144.v1.

The prediction in this study targets only the close price. Taking into account its values from day *t* − *N*, where *N* is a given number of days back, the task is to forecast its value at day *t* + 1. The training data set is therefore comprised of *N* independent variables *c*_*t*−*N*_, …., *c*_*t*_ and a dependent variable *c*_*t*+1_ for every day *t* ∈ [*N*, *M*], where *M* is the number of days for training. The validation data set considers the samples for the days *t* ∈ [*M* − *N*, *M* + *P*], where *P* is the number of days for validation; the last *N* days from training are also referred, as a means to predict the first day of validation. The test collection contains those records with *t* ∈ [*M* + *P* − *N*, *M* + *P* + *Q*], where *Q* is the number of days taken for testing and the last *N* days from validation are taken for the same reason mention before.

According to the terminology in [2], the window length is thus equal to *N*, the rolling window is equal to 1 day and the predict length also to 1 day. While the last two parameters have implicitly set values, the optimal period for the window length *N* has yet to be established during experimentation.

### 2.2 Deep learning for stock prediction. State of the art

In what follows, we present the state of the art in literature as concerns only prediction from stock data and not from additional information (such as textual references). Also, we solely review those entries where a DL model was used.

The study [3] builds a DNN on the TAQ data set of the NYSE for the Apple 1 min high-frequency stock pseudo-log-returns. The prediction of the average price is constructed on the basis of additional features: current time, price standard deviations and trend indicators provided for the selected window size. Based on the test predictions, a trading strategy is proposed in order to practically assess the performance of the constructed model.

The paper [4] models high-frequency (at 5 min) intraday stock returns from the Korean market through a DL neural network, both on raw data and with the support of additional feature extraction techniques (principal component analysis (PCA), autoencoders (AE), restricted Boltzmann machines). The study also tests the complementarity between the DL and the classical autoregressive model.

The article [5] uses a LSTM layer regenerating each trading day to predict the stock price movement from information recorded at 15 min from the Brazilian exchange. The attributes consist of the open, close, high, low and volume data and other technical indicators: future price, trading volume, intensity of the current movement tendency. The problem is transformed into a classification formulation, where there is a class indicating the increase of price and another its decrease. A trading operation is also simulated on the base of the predictions for the close price and compared to baseline strategies.

In [6], a cascade of three methods is used to predict daily stock moving trends from several indexes, each taken from the markets in one of the following countries: China, India, Hong Kong, Japan and USA. A wavelet transform first eliminates the noise from the input time series. Stacked AE then produce more general features and these are given to a LSTM. The initial features consist again of those of the trading data (open, high, low and close prices), of the technical variables (moving averages, commodity channel index, momentum indexes) and of the macroeconomic attributes (US Dollar index, inter-bank offered rate). Following the predictions of the model, a trading strategy is implemented on the index future contracts and compared to a traditional buy & hold procedure.

The paper [7] decomposes daily prices from Yahoo! Finance trading data into frequency components through a state frequency memory recurrent NN and combines the discovered patterns into the eventual stock price prediction.

In [8], 36 variables referring the daily open, high, low, close prices and technical attributes (momentum, moving average, psychological line among others) from the Google stock multimedia are modelled by a combination of a (2D)2PCA and a DNN.

From the S&P 500 ETF data, the study [2] takes into account the log returns for close price and trading volume as the stock time series variables, and stock chart images as additional information, all taken at 1 minute. A LSTM models the numerical attributes and a CNN mines the image data, while the features extracted from the two models are finally fused at the fully connected layer. A trading simulation is performed with the predictions on the adjusted close price of the proposed model against other strategies, including the classical buy & hold one.

### 2.3 Proposed LSTM and CNN architectures for stock data modelling

On the base of its conceptual formation and, as also seen before in literature, the first obvious choice for learning stock price series data is a LSTM. On the other hand CNN with 1D convolutions can also model temporal data. Moreover, it has been recently shown [1] for other sequential tasks, that CNN has many advantages over recurrent models: it creates a hierarchical representation over the inputs through its multiple layers, which identifies relationships within the data, and, computationally speaking, it allows a faster training.

The LSTM is a recurrent neural network that is able to implicitly learn long-term dependencies in the data [9]. This is possible through the structure of the repeating module that has several special components interacting with each other: a cell state and the three types of layers that control it—the forget gate that is in charge of knowledge that will be discarded from the cell state, the input gate that manages the information that will be kept, and the output gate that regulates what will be the output of the module.

The design of the LSTM selected for the current problem consists of the subsequent layer flow: several consequent LSTM layers, each followed by a Dropout layer, and a final Dense layer. The input data has the shape (*M*, *N*, 1), where *M* is the number of instances in the training collection, *N* is the window length and 1 is the number of indicators that are taken into account, i.e. in this case only the close price.

CNN can model data given sequentially over time through a special type of convolutional layer. The 1D layer of the CNN convolves the neural weights of each kernel with the input over a single temporal dimension. The input is thus taken with the shape (*N*, 1) for every of the M training samples given in this problem. The architecture of the CNN is disposed in the following manner: a sequence of 1D convolution layers of chosen kernel size and depth, each followed by a ReLU transfer layer for nonlinearity and a Max Pooling one, with Dropout in between convolutions. A Dense layer takes the flattened output of the last convolution, Dropout is inserted again and a final Dense one gives the output of the network.

Dropout layers were used in between the specific ones for regularizing both deep learning approaches [10], in order to prevent the overfitting generated by the available sample size.

A first step in evaluating the models is by fine tuning their parameters with the goal of minimizing the mean squared error (MSE) in a validation step. Subsequently, the architectures can be applied on a test set.

### 2.4 Post-learning trading strategy with hill climbing

A trading simulation is necessary in order to practically assess the performance of the learning models in identifying the trend patterns and leading to profit. Optimal rules for the trading strategy must thus be established. In the current work, we construct such rules by appointing two threshold parameters and determining their optimal value through a HC procedure.

There are other entries in the literature that make use of heuristic methods within trading strategies, but directed towards the popular evolutionary algorithms (EA). These are subsequently outlined. At the parameter level, the optimal values for variables of a filter rule are generated through EA in [11], namely the percent of price movement over which buy or sell decisions are taken, the number of hold days, number of delay days and the number of previous days taken into account. At the architectural level, the paper [12] comparatively studies two rule-based trading schemes involving several stock indicators: a neural network whose weights are found by EA and a genetic programming approach for generating the trading rule tree. The two trading strategy encodings are evaluated through different means incorporated into a single fitness function: profit in comparison to the buy & hold approach, penalty for those strategies that follow the buy & hold one, relation between profit and loss.

EA are indeed known for their optimization potential, flexibility and local optima circumvention. For the task at hand, however, there is no need for the complexity (in architecture and running time) of the EA. HC is a simpler, faster, yet efficient candidate for the rule parametrization desired herein.

Given *P* is the predicted close price for time *t* + 1 and *C* is the actual close price at time *t*, the pair of rules for the buying and selling operations are defined as in Eq (1).

$$\begin{array}{c} \left\{ \begin{array}{c} \;\text{if}\;P-C>\epsilon_{1}\;\text{then}\;\text{BUY}\\ \;\text{if}\;C-P>\epsilon_{2}\;\text{then}\;\text{SELL} \end{array}\right. \end{array}$$ (1)

where *ϵ*_1_ and *ϵ*_2_ are the threshold parameters that will be heuristically determined. In other words, the operation is BUY when the subsequent close price is predicted to be higher than the current close price above a certain threshold *ϵ*_1_, and it is SELL when the former is below the latter under a possibly different threshold *ϵ*_2_.

In our scenario, there is only one share per company involved, so SELL may occur only if the share is owned and BUY takes place only if it is not already bought.

The HC procedure starts with a random pair of values for *ϵ*_1_ and *ϵ*_2_ and, by means of mutation, it reaches a better configuration of parameter values. Mutation does not allow the values for the two variables to go lower than 0. The evaluation measures the gain on the validation set.

The gain is measured for a period [*t*_0_, *t*_*f*_]. At time *t*_0_ we implicitly have the initial investment in a share (BUY). A sequence of SELL-BUY-SELL-BUY-… follows, where the decision is taken according to the rules in Eq (1). The final operation is either a BUY or a SELL one. At day *t*_*f*_, the sum of gains and losses derived from the sequence of operations is computed as in Eq (2), where *n* is the number of operations, {*s*_1_, *s*_2_, …, *s*_*n*_} are the selling transactions, {*b*_1_, *b*_2_, …, *b*_*n*−1_} are the buying operations, *b*_0_ is the BUY at time *t*_0_, *b*_*n*_ is the last potential BUY and *C*_*k*_ the close price at operation *k*.

$$\begin{array}{c} S=\sum_{i=1}^{n}C_{s_{i}}-C_{b_{i-1}} \end{array}$$ (2)

The gain is defined as in Eq (3), where *S* is the sum of gains and losses obtained from trading as in Eq (2). If the user still has the share (last operation was *b*_*n*_), then the price of the share at final time *t*_*f*_ is added to the sum of gains and losses during trading.

$$\begin{array}{c} g=S+\{\begin{array}{c} 0,\;\text{if}\;\text{last}\;\text{operation}\;\text{was}\;\text{SELL}\\ C_{t_{f}},\;\text{if}\;\text{last}\;\text{operation}\;\text{was}\;\text{BUY} \end{array} \end{array}$$ (3)

Finally, a gain in percents is measured as shown in Eq (4).

$$\begin{array}{c} pg=\frac{g}{C_{t_{0}}}\times 100 \end{array}$$ (4)

The same procedure goes for the computation of profit for the test period. Two step-by-step scenarios are illustrated in Fig 8 in subsection 3.3.

The considered heuristic-based rule trading system is compared to two baseline methods, i.e. buy & hold and the Bollinger bands approach. These are considered as benchmark due to their traditional tactical use in practical stock trading and to their frequent employment in comparisons to newer methods, as found in literature (see section 2.2 on state of the art).

The buy & hold approach simply performs a BUY operation at time *t*_0_ and a SELL one at time *t*_*f*_; the gain is directly the difference $C_{t_{f}}-C_{t_{0}}$.

As concerns Bollinger bands, it is the bounce strategy that is employed for comparison. If the close price reaches the upper band then it will bounce back to the middle area (lower price), so the action should be SELL. In reverse, if the close price touches the lower band, the operation should be BUY, since it will bounce to the middle area (higher price), as well.

## 3 Results and discussion

Experiments target three directions:

1. **Parametrization**. On the one hand, the window length is a parameter of the time series task that controls both accuracy and processing time, and its value must thus strike a balance between the two. On the other hand, deep methodologies are known for their sensitivity to parameter tuning for every problem.
2. **Prediction power**. The accuracy of estimation of the LSTM and CNN is measured in comparison.
3. **Trading simulation**. The effect of the deep learning prediction is illustrated in a trading scheme, based on thresholds that are additionally heuristically determined.

The validity and utility of the framework will be consequently measured from multiple facets:

1. **Choice of the optimal window length**. The performance of the model concerning this parameter will be measured by comparing the MSE results on the validation data when a minimal number of reference days back (30) is used with the best ones achieved when the length is taken from a manual search. A Wilcoxon-Mann-Whitney statistical test will be employed to compare if the difference in performance between the results of 30 runs is significant as to justify an increased running time.
2. **Architecture of the deep learners**. The architecture of the LSTM and CNN will be manually selected to handle all performance issues, i.e. overfitting, running time and accuracy. When the difference in mean accuracy from 10 repeated runs between two architectures is not significant, it will be opted for the least complex one.
3. **Comparison of deep prediction results**. The mean accuracy for MSE obtained by the LSTM and the one of the CNN will be compared. Standard deviations with the minimum and maximum will also be calculated. Should the mean results be close, then the Mann–Whitney U test will determine if the difference in the MSE results from 10 validation runs is significant. Besides these, the MSE of the method in [6] is considered for comparison.
4. **Gain measurement from a trading scenario**. The gains in percents obtained from a simulated trading on the test data, on the base of the deep learning prediction results and the heuristic trading scheme, will be illustrated as bar and box plots. They will be compared in mean, minimum, maximum and standard deviation with the ones of the traditional trading tactics of buy & hold and Bollinger bands. The gain amounts derived from the two deep learners will also be put against those of the classical schemes. Again, if the mean results are very close, the same statistical test will confirm if there is a significant difference or not.

In order to conduct an objective statistical analysis of the performance measures, the data was split into training, validation (for parameter tuning and HC application) and test sets and the Monte Carlo cross-validation with 10 repeats was performed when reporting the accuracy results. Training contains 60% of the records for each company, validation takes the following 20% and test holds the final 20% instances.

It is well-known that deep learning presents a big problem as concerns the large running time necessary for training. The running times for the CNN and LSTM favor the former by a large extent. In average over all 25 companies, the building of the CNN model, when an architecture with two convolutional layers is used, takes 35.22 seconds. Comparatively, the corresponding value for the LSTM, having also 2 specific layers, takes 114.29 seconds and 160.91 seconds respectively for one with 3 such layers. The reduced running time for CNN allows for a more detailed parameter tuning. As the evaluation of one parameter setting for a company takes around 35 seconds, considering all 25 would lead to a running time of almost 15 minutes and this would not allow for the evaluation of too many combinations. Consequently, one company is next selected as representative for parameter tuning, and the discovered settings will be further used for every company, although it is natural that the found values are not necessarily optimal overall.

### 3.1 Parameter tuning

**Fig 3:**
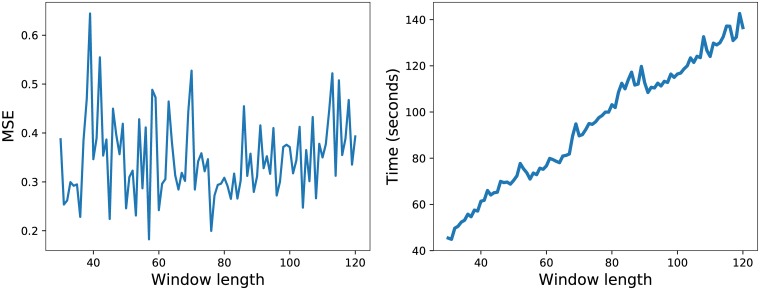
The average validation MSE and training running time of the CNN when the number of days back is varied between 30 and 120 for the BRD symbol.

One important parameter that directly affects the running time regardless of the employed model is the window length. The company randomly chosen is the one with the symbol ‘BRD’ and different window lengths are tried between 30 and 120. For each case, a CNN is used to train the model, then it is applied for the validation set and the MSE is computed. The first plot in Fig 3 illustrates the average validation MSE as obtained after 10 repeated runs of the CNN model. Naturally, smaller values are preferred. The second plot shows the training running time in seconds when the same window length is varied.

The best result in the first plot corresponds to the window length 57. Since there is a high running time gap between 30 and 57 in the second plot, the two settings are further more thoroughly investigated through statistical means. The question that arises is whether the MSE results derived from the two window lengths are significantly different. The CNN is repeated for 30 times for each of the two window lengths, the average validation MSE results are of 0.41 and 0.36 and the two MSE vectors are next compared via a Wilcoxon–Mann–Whitney statistical test. The calculated p-value of 0.26 indicates that the two settings do not lead to statistically different results and the smaller window length of 30 is further preferred in all experiments because of the lower running time.

**Fig 4:**
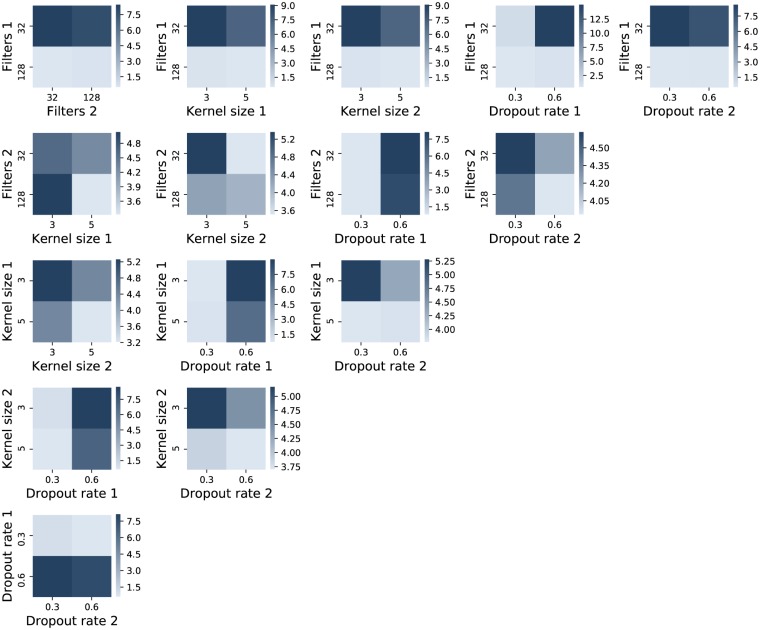
Average approximated validation MSE values from 10 repeated runs for the combination of various parameter values for a CNN with two layers. Lighter color stands for lower MSE.

**Fig 5:**
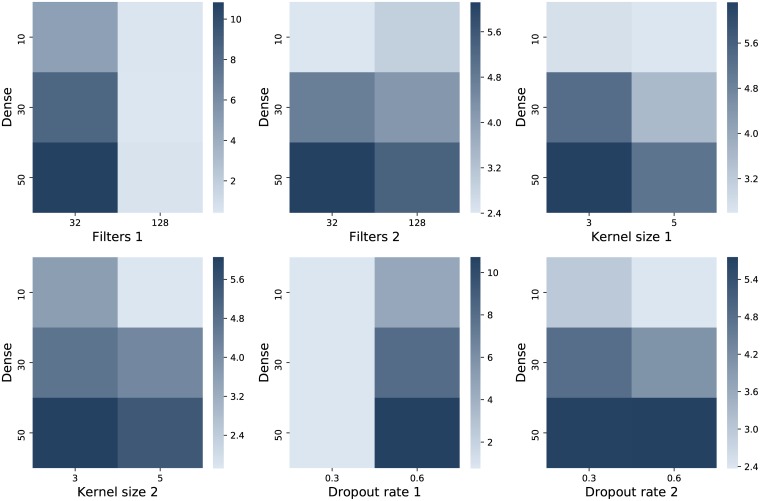
Average approximated validation MSE values from 10 repeated runs for the combination of the number of dense units as combined with various parameter values for a CNN with two layers. Lighter nuances signify lower MSE.

**Fig 6:**
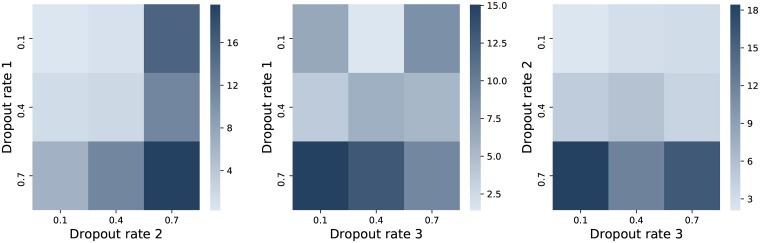
Average approximated validation MSE values from 10 repeated runs for the combination of dropout rates values, as applied after each of the three layers. Lighter coloring denotes a lower MSE.

The next step of parametrization concerns the deep approaches. There are two general architectures tried for the CNN: with two and with three convolutional layers. For each of them, the convolutional kernel parameters and the dropout are tuned. Firstly, the approach with two layers is considered and various values for parameter combinations are tried. In all the cases there are 10 repeated runs considered, where the averages are computed, and the data used for this purpose consists only of the stocks for symbol ‘BRD’. Subsequently, the presented results are calculated by fixing the parameter combinations two by two and the outcome is obtained by averaging the results for all the other varying involved parameters. The numerical values that appear in the labels for Figs 4, 5 and 6 refer to the number of the layer where that specific parameter appears. Lighter colors correspond to better results.

Fig 4 shows the combinations between the pairs of values for filters, kernel sizes and dropout rates for the CNN with two layers. A result that consistently remains better is achieved for the larger number of filters and the lower value of the dropout rate for the first layer. The numbers of filters for the second layer leads to better results also for the value 128, although the gap between the two options is not as high as it was for the first layer. The kernel sizes are taken 5. As mentioned above, the dropout rate for the first layer is taken 0.3, while the one for the second layer appears to conduct to better outputs for 0.6.

Fig 5 investigates 3 distinct options for the number of units from the dense layer that is employed after the flattening layer. The best choice is 10, while the interaction with the other parameters only further confirms the previous choices.

Alternatively, a CNN with three layers is also tried. The parameters found to be appropriate for the version with two layers are adopted in this case, except for the dropout rates. The number of filters for the third layer is taken 256 and the kernel size is set to 3. Fig 6 shows the interaction between the 3 dropout rates considered, each with 3 possible values. As observed, the nuances indicate better results for small amounts in first two dropout rates and generally good for the last one in all cases, with only a preference for 0.4 in the middle plot, where the rates for the first and third layers are confronted.

By comparing the best results illustrated by means of lighter nuances (and observing the minimum values from the color bar on the right) in Figs 4 and 5 to the ones in Fig 6, it can be concluded that the CNN model having two layers performs similar or even better in terms of MSE values. Another argument for keeping the 2 layer model for the next experiments is given by the running times, as this takes 44.26 seconds to build it, while training the one with 3 layers lasts 106.05 seconds.

The parameters of the LSTM were chosen manually with 50 units in each specific layer and a dropout of 0.2. The high running time did not allow a similar thorough parameter tuning for this model. However, alternatives with 2 and 3 layers respectively were tried. Naturally, the higher the number of layers, the greater the running time is. When it comes to MSE obtained over all the 25 companies, the variant with 3 layers had an average of 0.92, while the simpler alternative led to a considerably higher average of 2.39. Accordingly, the 3 layers option is preferred next.

### 3.2 Prediction results

Table 1 illustrates the MSE average results over all 25 companies on the validation set as obtained by the CNN and LSTM from 10 repeated runs. The running times (in seconds) that were necessary for training the two deep learning models are in the second row of the table and those for building the HC used in the next experiment are given in the last row. The HC uses for the evaluation of the potential solutions the previously saved models of CNN and LSTM. It can be seen that the CNN needs approximately 4.5 less time than the LSTM, however at a somewhat high MSE difference.

**Table 1:** Comparison between CNN and LSTM architectures in terms of MSE on the validation set, running time for training them (time 1) and running time for the HC when using the previously saved CNN and LSTM models (time 2). All results are reported in average over 10 repeated runs of the method.

Measure	CNN				LSTM			
	Mean	Min	Max	SD	Mean	Min	Max	SD
MSE	40.63	3.9E-06	981.97	192.23	0.92	1.5E-06	14.51	3.1
Time 1	35.22	12.58	71.41	13.45	160.91	59.88	223.94	53.9
Time 2	126.5	43.43	186.8	44	179	60	259.5	62

**Fig 7:**
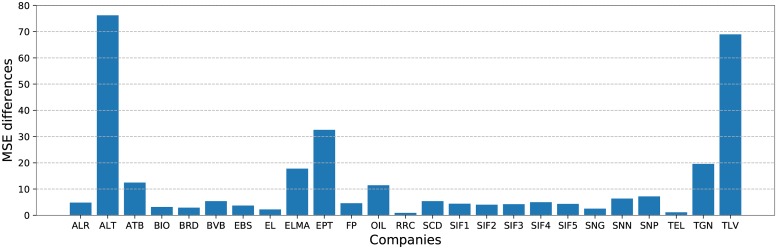
The number of times MSE for CNN is larger than the one obtained by LSTM for each company in turn.

Although the difference between the mean MSE for the 2 models appears to be rather substantial, the gap between them is not that large for all companies. Each bar in Fig 7 illustrates the ratio between the MSE for CNN and LSTM for each company in turn. Although for ‘ALT’ and ‘TLV’ the discrepancies appear to be massive, they are obtained in both cases from the digits after the decimal point. The differences in the mean values in Table 1 come from ‘TGN’, which gives the maximal values for both and also has the highest close price values in Fig 2, and from the next largest values in both cases, for ‘EBS’, where CNN reaches a MSE of 29.39 and LSTM one of 7.07, respectively.

In order to compare the proposed deep learning architectures to the state-of-the-art methodology in [6], the latter was implemented and applied in the current study. In short, there are 11 more technical indicators used, i.e. moving average convergence divergence, commodity channel index, average true range etc; then a stacked autoencoder is employed to generate deep high-level features which are fed to a LSTM to forecast the close price. The obtained results were better than the ones from CNN from Table 1, but weaker than the ones of the pure LSTM. The mean MSE was of 7.69, while the corresponding average running time 1 was of 263.63 seconds, considerably larger than the one of the LSTM, which was already relatively high.

Since the predictors in [6] led to promising results in the indicated research paper, the same features are additionally used for the LSTM with the aim of forecasting the close price. The mean MSE reached a value of 1.55, higher than the 0.92 reported in Table 1 and a running time for training of 298.62 seconds. Since both results are weaker than the current LSTM that uses the previous 30 values only for the close price, the multiple predictors are omitted next.

### 3.3 Example trading scenario

The predictions of the deep learning approaches are subsequently used in a trading simulation in order to effectively test the performance of the models.

All trading scenarios start from the assumption that one share is bought at the beginning of the trading period and then, depending on the strategy, it can be sold at some point in time, bought again later and so on. If at the end of the test period the share is still owned, it is evaluated at this final time as if it was sold just then. For each company, the gain is computed by subtracting the initial value of the share. The sum, the average, as well as minimum and maximum over gains are reported for all companies in order to assess the overall efficiency. The sum over all the initial values for every one share of all companies is of 555 RON (Romanian currency), i.e. 117 euro at the time of writing, so that can be viewed as the initial investment.

The HC algorithm in charge of setting the optimal thresholds for buying and selling runs for 60 iterations. 5 restarts are used in order to have higher chances of escaping local optima. The candidate solution encodes the *ϵ*_1_ and *ϵ*_2_ in (1). The two values are bounded to the interval given by 0 and one quarter of the difference between the maximum and the minimum value for the close price over the validation period. Surely, the high value of this interval is different from one company to another. A mutation with normal distribution is used and the mutation strength is set as the quarter of the maximum value *ϵ*_*i*_ can have, *i* ∈ {1, 2}. The two values found by the HC are the ones deemed as very suitable for the validation set, meaning that the gain on this period is maximized. There are 2 HC versions, one that uses as fitness evaluation the CNN approximation of the close price and one that employs the LSTM for the same purpose. They are further denoted as HC-CNN and HC-LSTM. Subsequently, the solutions reached for each company in turn are used for the test period and the gains are computed. For overcoming the stochastic nature of the HC results, the reported outcomes are averaged over 10 repeated runs.

**Fig 8:**
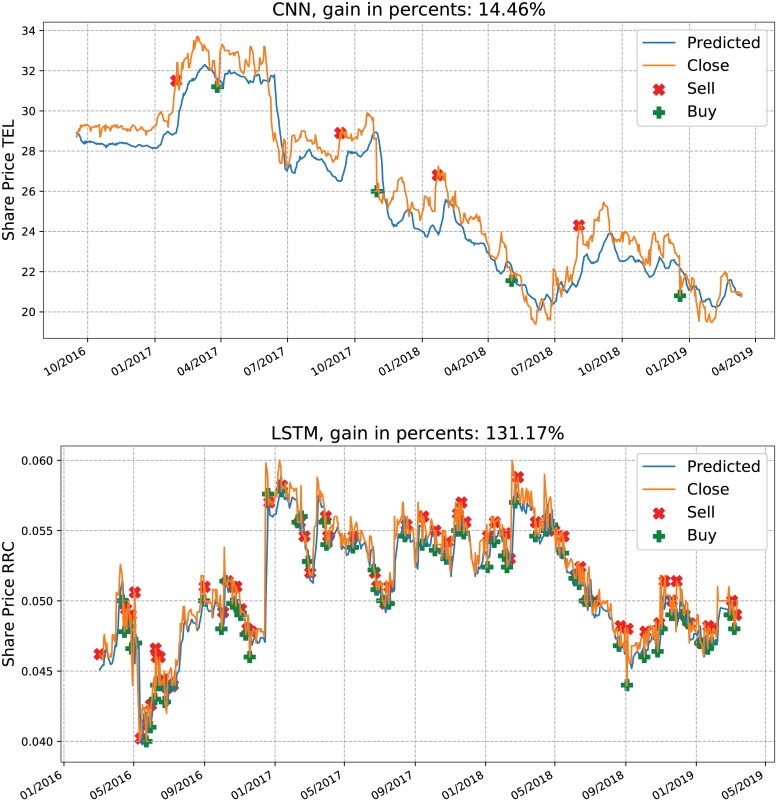
Scenarios for the test period using CNN with *ϵ*_1_ = 0.38 and *ϵ*_2_ = 2.31 for TEL and LSTM with *ϵ*_1_ = 3.3E-05 and *ϵ*_2_ = 4.4E-04 for RRC. While for the top case the share is sold 4 times within the period, for the other case it is sold 58 times.

Fig 8 illustrates the close price and the predicted one for the test period for two companies that should be illustrative of the process. The top plot shows the behavior of the CNN model for ‘TEL’ with the thresholds found as optimal by the HC in validation, which are *ϵ*_1_ = 0.38 and *ϵ*_2_ = 2.31. The red *x* and the green + signs indicate when the stock share is sold or bought, respectively. Although, when comparing the input share price with the output, the price decreases from the beginning of the test period till the end, the proposed model reaches a gain in percentage of 14.46%. In fact, for this company the CNN-based model is the only one that has a gain, while the others register losses. The LSTM has a positive outcome for this company only in 1 out of 10 HC runs (when it was a gain of 7.67%), while the CNN registers gains reaching from 2.09% up to 15.85% over the 10 repeated runs.

The second plot uses the thresholds discovered by HC-LSTM for the company with the symbol ‘RRC’, which are very close to zero—*ϵ*_1_ = 3.3E-05 and *ϵ*_2_ = 4.4E-04. The predicted prices and the found thresholds determine a high number of transactions and lead to a gain of 131.17%. Nevertheless, the share price is very low and such a great gain in percents leads in fact to a smaller gain in actual money value as opposed to the real money amount obtained, corresponding though to a smaller gain in percents, of the CNN in the top plot.

**Fig 9:**
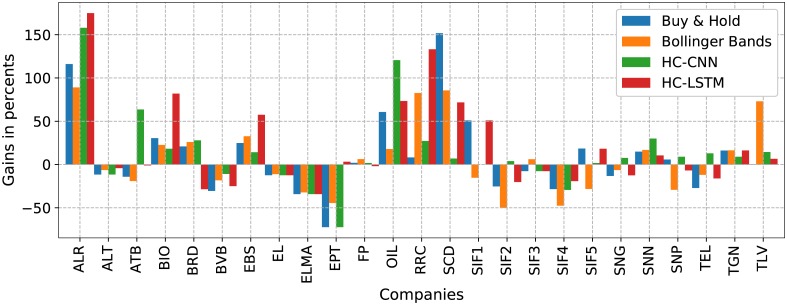
Gains in percents reached by each of the 4, HC-deep or traditional, tried scenarios for every company in turn.

Fig 9 shows the gains in percents for all 25 companies, involving the HC-deep schemes and the traditional scenarios of buy & hold and Bollinger bands. As mentioned above, the outputs for HC-CNN and HC-LSTM are the results of a mean over 10 runs of the HC. For all the others, the results are deterministic, so multiple repeated runs are not necessary. A similar plot for gains in money is not plotted because the differences in the price for the shares of the different companies would flatten the bars for most of them and only those with higher values would dominate the figure.

Table 2 shows the mean, median, sum, minimum, maximum and standard deviation for different types of results over the tried scenarios and for all the 25 companies. Two more financial quality measures are added to the results, i.e. annualized return (AR) and sharpe ratio (SR). The first one shows the gain earned by an investment over a given time period, but reported to one year. Eq (5) illustrates how AR is calculated; SP and BT stand for selling and buying price respectively, while days represent the number of days between BP and SP. Eq (6) shows how the SR is computed. DR denotes daily return, while *avg* and *std* represent average and standard deviation, respectively.

$$\begin{array}{c} AR=100\cdot [{\left(1+\frac{SP-BP}{BP}\right)}^{\frac{365}{days}}-1] \end{array}$$ (5)

$$\begin{array}{c} SR=\sqrt{252}\cdot \frac{avg(DR)}{std(DR)} \end{array}$$ (6)

**Table 2:** Comparison between the 4 distinct scenarios with respect to the gains in percents and values, the number of times one share is sold, annualized return and sharpe ratio. Results for HC-CNN and HC-LSTM are averaged over 10 repeated runs.

Measures	Buy & Hold	Bollinger Bands	HC-CNN	HC-LSTM
Gains on the test data in percents				
Mean	9.76	6.2	12.97	20.37
Min	-72.4	-50.12	-72.4	-34.19
Max	151.78	89.11	170.62	175
St. Dev.	46.28	40.2	43.21	49.74
Gains on the test data as values				
Sum	60.88	80.19	53.6	**112.43**
Mean	2.43	3.21	2.14	4.5
Min	-10	-6	-1.44	-8.4
Max	47.5	48	25.33	74.5
Times share sold				
Mean	1	5.8	3.62	7.37
Min	1	2	0	0
Max	1	11	49	50.2
St. Dev.	0	2.24	9.38	12.39
Number of times out of 25 when gains were positive				
Times	13	12	**17**	12
Annualized return on the test data				
Mean	0.49	1.82E+06	8.57E+13	3.58E+12
Median	1.19	28.48	29.43	757.4
Min	-23.49	-15.33	-52.08	-13.03
Max	24.22	4.5E+07	2.14E+15	8.95E+13
St. Dev.	11.73	8.9E+06	4.2E+14	1.75E+13
Sharpe ratio on the test data				
Mean	0.14	0.59	1	-0.33
Median	0.17	0.17	0.41	-0.09
Min	-0.91	-1.46	-4.78	-8.21
Max	0.96	3.99	10.91	1.78
St. Dev.	0.43	1.33	2.76	2.07

AR shows what an investor would earn over a period of time with respect to the annual return, without any indication of the volatility for the investment. Its output can reach very high values when the stock is bought at a low price and sold at a significantly higher price in a very short period of time. This observation becomes obvious, if we see in Eq (5) that the exponent is obtained by dividing 365 to the number of days between the buying and the selling dates: the smaller the number of days between the two dates, the higher the power exponent becomes and, consequently, the higher the entire AR. This is the reason why the AR values for the buy & hold in Table 2 are always small (the two dates represent the first and the last days). For the other three options, various inspired speculations led to very high values for some of the companies. For this measure, the median is more relevant than the mean. For this measure, HC-LSTM appears to point to the most inspired times for buying and selling.

In contrast to the AR, SR takes into account the volatility of the investment returns. Since the volatility is generally high for the companies in the current data set, and it represents the denominator, the values for SR are generally low. The highest value is registered for HC-CNN both for mean and median, in line with the results holding the number of times where gains were positive.

**Fig 10:**
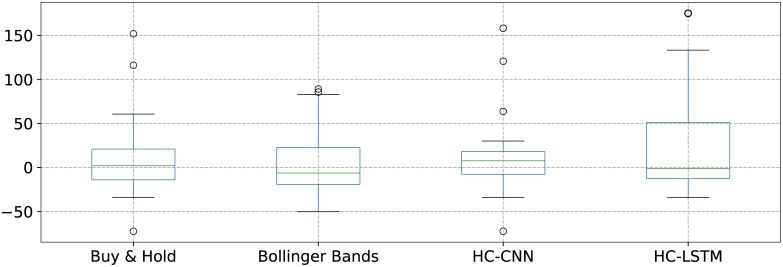
Box plots with the gains in percents for every scenario in turn as computed over all companies.

**Fig 11:**
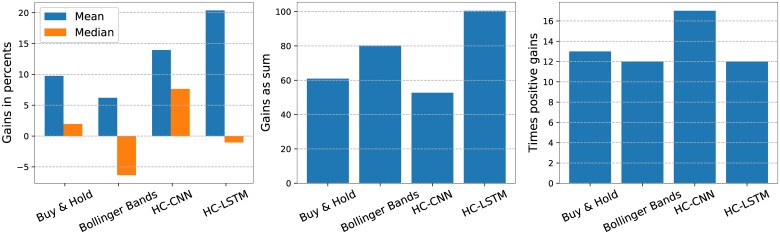
Results obtained over all companies by each of the tried scenarios. Mean and median of the percentage gains over all companies when having a share at the beginning of the test period and applying the corresponding scenarios are shown in the left plot. The middle plot shows the gains in terms of money for every scenario, while the right one illustrates the number of times out of 25 possible in which the trading led to a positive gain.

Fig 10 shows box plots with the gains in percents over all companies for both the deep-based and classical scenarios. The HC-CNN has the smaller inter-quartile range, while the HC-LSTM one has the largest variability. The highest median is also obtained by the HC-CNN model, fact that can be better observed in the first plot from Fig 11. The box plots with the largest parts of the inter-quartile ranges on the positive side are the ones corresponding to the HC-CNN and HC-LSTM models. What is more, most of the box that defines the inter-quartile range for HC-CNN is on the positive side. This also corresponds to the fact that it has the highest number of times out of 25 when the gains on the test period are positive, as shown on the last row of Table 2, as well as in the third plot from Fig 11. It comes as a surprise however that the largest gain in value (i.e. in RON) is achieved by the HC-LSTM, as indicated in the middle plot from the same Fig 11, despite the smaller number of times in which the gains were positive. This can be explained by the largest mean value for the gains on the test data in percents in Table 2, but also by the fact that the better results are obtained for companies that have more valuable (i.e. higher priced) shares. The highest value for the AR measure also indicates that HC-LSTM speculated well the times for buying and selling.

Naturally, the buy & hold and Bollinger scenario do not need a validation period, since they do not use any automatic learning or calibration of the models. In this respect, their gains by applying the scenarios on this period would only provide further tests of the models. An interesting difference between the validation and test periods is given by the fact that the buy & hold strategy reaches a positive outcome for 16 companies for the former, as compared to only 13 in the period used for test, despite a relatively similar percentage gain of 9.91% and 9.76%, respectively. This indicates that there are more companies that had their shares with values rising in that period and also puts the buy & hold strategy on the top position among the two deterministic options discussed for this period with a gain of 124.99 RON. The Bollinger model gets only 74.93 RON. The HC-CNN and HC-LSTM are certainly far better in this period, since the goal of the HC was to find proper values for the thresholds by having access to the validation data. Their gains are of 202.65 and 239.38 RON, respectively, with 22 and 24 companies for which the gain is positive.

## 4 Conclusions and future perspectives

The paper puts forward two deep learning models for stock price prediction in the Romanian market, alongside this new data collection. The estimations of the two architectures are used within a trading strategy. The optimal amount of difference between the close price of the current day and the predicted price for the next day towards deciding BUY and SELL operations is determined through a HC procedure. It is interesting to see that the two deep networks lead each to a distinct facet of the gain within the trading simulation: while the LSTM has a higher gain in terms of the sum of money earned, the CNN has a higher number of times gained than lost for the 25 companies watched. Also, while HC-LSTM reaches a better value for the annualized return, HC-CNN leads to a better sharpe ratio.

Future work aims to target enhancements both at the level of the learning and that of the optimization. Other types of recurrent architectures, such as echo state networks, can be tried. The deep models (LSTM, CNN) can be more elaborately parametrized as in [13] and ensembles similar to [14] can be constructed with traditional machine learning techniques, i.e. SVM [15] or random forest [16]. The landscape of multiple stock indicators can be examined by EA [17] in order to work with several selected predictors. Also, the parameters of the trading strategies can be appointed in an evolutionary fashion. More complicated rules can be evolved by multiple population EA [18] in comparison to the genetic programming and neuro-evolution approaches in the state of the art. Additional textual knowledge that may predict the rise or drop of the stock price of a company triggered by its appearance in the media [19] are planned to be also investigated as auxiliary input. Also, more sophisticated scenarios like allowing to buy more stocks or having a sum and investing in various companies (not necessary all of them) in order to increase the overall gain will be also tried within the future directions.
